# Editorial: Insights in inflammation: 2022

**DOI:** 10.3389/fimmu.2023.1224343

**Published:** 2023-05-31

**Authors:** Pietro Ghezzi, Rudolf Lucas, Simone Mader, Pierre Miossec, Sandra Sacre

**Affiliations:** ^1^ Department of Biomolecular Sciences, University of Urbino, Urbino, Italy; ^2^ Medical College of Georgia, Augusta University, Augusta, GA, United States; ^3^ Institute of Clinical Neuroimmunology, Biomedical Center and University Hospital, Ludwig-Maximilians University Munich, Martinsried, Germany; ^4^ Immunogenomics and Inflammation Research Unit, Edouard Herriot Hospital, Hospices Civils de Lyon, Lyon, France; ^5^ Department on Clinical and Experimental Medicine, Brighton and Sussex Medical School, Falmer, Brighton, United Kingdom

**Keywords:** inflammation, signallig, musculoskeletal diseases, allergy, transcription factors

This Research Topic is focused on new insights, novel developments, current challenges, latest discoveries, recent advances, and future perspectives in the field of Therapeutics. Most manuscripts in this section deal with the inflammatory component of disease which include specific musculoskeletal disease (osteoartritis, ankylosing spondylitis), other specific conditions (nasal polyps, asthma, atopic dermatitis, liver ischemia/reperfusion, burn and sepsis). The pathways and mechanisms investigated were related to signalling mechanisms (STAT3, FK506-binding proteins), inflammatory cytokines, receptors (prostanoid receptor CRTH2 and the scavenger receptor MSR1), transcription factors (Nrf2 and HIF-1a) and processes related to cell death (ferroptosis, autophagy, efferocytosis).

The cytokine signalling pathway involving JAK/STAT is a validated pharmacological target and JAK inhibitors are now approved for some chronic inflammatory disease. Yang et al. review the role of STAT3 in liver ischemia/reperfusion injury describing a not widely known effect on lipid metabolism. Tomiaki et al., using FK506 as a tool in a model of allergen-mediated airway inflammation, investigate the signalling mechanism of group 2 innate lymphoid cells (ILC2s) and type 2 helper T (TH2) cells in asthma.

A review by Gudgeon et al. highlights the importance of Macrophage scavenger receptor 1 (MSR1) as a potential biomarker and pharmacological target in inflammatory disease and cancer. This is one of the scavenger receptors that facilitate the uptake of lipoproteins by macrophages and hence the formation of foam cells. The review discusses MSR1 mutations, transcriptional regulation and signalling pathway and puts these in context with its possible role in innate immunity, atherosclerosis and diseases associated with inflammation.

A research paper by Chen et al. studied the expression of chemoattractant receptor-homologous molecule expressed on Th2 cells (CRTH2) in nasal polyps and found an association with eosinophil infiltration, which could be important as CRTH2 antagonists are being tested in clinical trials for other disease conditions.

Wang and He review the anti-inflammatory mechanisms mediated by Nrf2, and its interaction with pro-inflammatory transcription factors such as NFkB, in the context of osteoarthritis. This is a research field that may have clinical relevance given the poor response of osteoarthritis to TNF neutralizing agents and the fact that Nrf2 activators are already marketed for other pathologies (e.g. dimethylfumarate in multiple sclerosis). In a research paper, Sun et al. investigate the therapeutic effect of cold plasma in a mouse model of atopic dermatitis as well as in human keratinocytes *in vitro*. Their study identifies a mechanism by which cold plasma treatment induces the protein mesencephalic astrocyte-derived neurotrophic factor (MANF) that would then reduce inflammation and endoplasmic reticulum stress, possibly by increasing HIF-1 α at the transcriptional level.

Long et al. performed a pre-registered systematic review with meta-analysis of studies on the efficacy of iguratimob, an anti-inflammatory drug approved in China and Japan, on ankylosing spondylitis. The results indicate an efficacy of the drug in association with standard therapy. However, using a risk-of-bias tool they find that the evidence was judged to be moderate to very low, which should encourage clinicians to perform higher-quality trials.

Another approach represented in this Research Topic is that of genetic association. Liu et al. studies the association of variants in the IL-17 family genes with susceptibility to human diseases and finds an association with several diseases, including musculoskeletal disease, asthma and cancer. This study may be clinically relevant as anti-IL-17 antibodies are already approved for some pathologies.

Three reviews dealt with cell death. Zhang et al. reviews ferroptosis, a form of cell death regulated by iron, with a particular focus on osteoclasts and osteoblasts and its potential relevance in musculoskeletal disease. The review by Saas et al. deals with efferocytosis, a form of phagocytosis of apoptotic neutrophils by macrophages. They describe the various molecular mechanisms of efferocytosis and its role as an active mechanism involved in the resolution of inflammation. Autophagy is a process that is frequently, but not necessarily, associated with cell death and consists of the degradation of cellular components. Zhao et al. discuss the molecular mechanisms and pathways that regulate autophagy with a focus on sepsis-induced acute kidney injury.

Finally, research by Mulder et al. studies in depth the inflammatory response in burn wound tissue (eschar) from patients. By using immunohistochemistry, flow cytometry and immunoassays, the authors characterize the local infiltration of immune cells in the wound and correlate it with the expression of several cytokines.

## Author contributions

PG wrote the original manuscript. All authors edited and approved the manuscript.

